# Public Perceptions of Rotator Cuff Tears

**DOI:** 10.3390/clinpract14030058

**Published:** 2024-04-25

**Authors:** V. V. N. Manohar Devarasetty, John E. Kuhn, Eric N. Bowman

**Affiliations:** 1 School of Medicine, Vanderbilt University, Nashville, TN 37232, USA; veera.devarasetty@vanderbilt.edu; 2 Department of Orthopaedics, Vanderbilt University Medical Center, Nashville, TN 37232, USA; j.kuhn@vumc.org

**Keywords:** rotator cuff, tear, injury, shoulder, perception, public understanding

## Abstract

(1) Background: Full-thickness rotator cuff tears (RCTs) impact 25% of those over 60 and 50% over 80; however, minimal data exists on public understanding; (2) Methods: The primary outcome was to determine the public’s baseline understanding of RCTs utilizing a 36-question survey regarding anatomy and function, risk factors, diagnosis and treatment options, and expectations. Secondarily, we evaluated the effect of an educational video and informational handout created by the authors to improve understanding. Participants ≥ 18 years were recruited from the senior author’s clinic and online discussion platforms over a 5-month period; (3) Results: Baseline surveys were completed by 382 individuals: 56% men, 64% Caucasian, 27% with at least a master’s degree, and 56% with very little or no RCT knowledge. Mean correct answer scores improved from 47% to 68% posteducational intervention (*p* < 0.001). Males, higher education level, healthcare experience, and a higher self-rated understanding of RCTs were significantly correlated with higher survey performance (*p* < 0.001); (4) Conclusions: The public’s knowledge of RCTs at baseline was poor, with demographic factors correlating with survey performance. The educational intervention effectively enhanced participants’ understanding. By focusing on common misconceptions, this data can help clinicians align patient expectations and enhance patient outcomes.

## 1. Introduction

Rotator cuff tears (RCTs) are one of the most common orthopedic conditions, with a reported prevalence varying from 22 to 39% [1,2]. The incidence of full-thickness RCTs has been increasing over the past few decades proportionally with the aging population in the United States, affecting 25% of individuals in their 60s and 50% of individuals in their 80s [3,4]. They are more likely to occur in the dominant arm of older patients who have a history of smoking, hypercholesterolemia, and diabetes mellitus. RCT treatment is determined based on each patient’s age and functional impact on their employment and recreational activities. Conservative treatment includes physical therapy, nonsteroidal anti-inflammatory drugs (NSAIDs), and steroid injections, which are initiated before surgical management for degenerative tears [2,3,4]. Surgical repair of RCTs is very common, with an incidence as high as 165 repairs per 100,000 person-years and increasing by 1.6% per year [5,6,7].

Despite the prevalence and impact of RCTs, there is limited research on the public’s knowledge of this condition. The primary objective of this study is to explore the current state of public knowledge regarding RCTs, identify gaps in understanding, and discuss the implications of these findings for clinical practice and public health. Secondarily, we aimed to assess the impact of an educational video and informational handout in enhancing baseline knowledge. We hypothesized that the public’s baseline knowledge would be low, defined as less than 50% correct responses on basic information regarding diagnosis, treatment, and outcomes. Such misunderstandings could lead to unrealistic expectations and negatively impact the patient−provider relationship. We believe that by synthesizing the public’s knowledge about RCTs, this data will provide valuable insights to develop effective educational strategies to align patient expectations and ultimately improve patient outcomes.

## 2. Materials and Methods

This cross-sectional study was approved by our Institutional Review Board (IRB #220909). A 36-question online survey was created that sought to assess the various aspects of the public’s understanding of RCTs. Questions were formulated from a literature review on different aspects of RCTs and were modified by the consensus of two sports medicine fellowship-trained surgeons [1,2,3,4,8,9]. The survey was developed following standard principles of item generation, item reduction, questionnaire formatting, and pretesting [10]. Item generation identified 43 questions that would be reasonable to include in the survey. To reduce the question burden and select only a subset of items for the survey, item reduction was implemented. This process resulted in the survey including 36 questions, with the majority being multiple-choice. This questionnaire was pretested with a total of 11 participants with a range of 19 to 63 years of age and varying levels of medical knowledge. The respondents completed all the questions in the questionnaire, and there were no indications of bias or offensiveness in their feedback. However, based on their comments and the two senior authors’ input, the questions and responses were further refined.

Participants were recruited using a flyer (see File S1) at the senior author’s orthopedic practice and a personalized message (see File S2 for example) posted on a variety of social media (Instagram, Facebook) and subreddit forums (r/nba, r/baseball, r/nfl, r/collegebasketball, r/mlb, r/golf, r/hockey, r/askreddit, r/casualconversation, r/sportsmedicine, r/athletictraining, and r/physicaltherapy). Participation in the online survey assessing their “understanding and management of rotator cuff tears” was voluntary. Responses were collected over a 5-month period from October 2022 to February 2023. The only criteria for study inclusion included being over the age of 18. Each participant completed the survey anonymously without the influence or assistance of any of the co-authors. No compensation was provided for the completion of the survey.

Study data was collected and managed using the research electronic data capture (REDCap) database, a secure, web-based software platform designed to support data capture for research studies [11,12]. All components of the questionnaire were written to an 8th-grade reading level [13]. The questionnaire was primarily designed using a multiple-choice format, with several questions utilizing a Likert scale. Other questions required a simple “yes” or “no” response, while some required the respondents to circle a number on a scale of 1 to 10. The survey (see File S3) was organized into seven sections (Table 1). There were six questions about demographics that asked participants about their sex, race/ethnicity, education level, whether they worked in the medical profession, whether they or someone they knew had been diagnosed with an RCT, and how they rated their knowledge of RCTs. Additionally, there were three questions about the anatomy, function, and epidemiology of RCTs, followed by three questions about the risk factors for RCTs. The survey included eleven questions regarding the diagnosis and treatment options for RCTs, as well as four questions about the surgical management of RCTs. Finally, there were four questions about the risks of RCT surgery and five questions regarding postoperative expectations of RCT surgery. Of the 36 questions in the survey, 17 were considered factual, having a single answer that is deemed to be correct from current orthopedic literature (e.g., “Rotator cuff tears are most common in what age group?”). The remaining 19 questions did not have a single correct answer and sought to determine the participants’ perceptions. After completion of the baseline survey, participants were directed to an educational intervention that presented participants with a comprehensive video and a transcript covering all aspects of RCTs and their management (see File S4). The information for this educational intervention was obtained from patient-directed, peer-reviewed literature [1,2,3,4,8,9]. After reviewing the educational material, participants were instructed to complete a postsurvey to gauge the intervention’s impact.

### Statistical Analysis

A power analysis was not conducted initially to determine a study size due to the cross-sectional nature of this study. However, a posthoc power analysis was performed to assess the study’s statistical power retrospectively. The results revealed that the study possessed a power exceeding 99% to detect differences between pretest and posttest scores when stratified by participant demographics and question categories. Descriptive statistics were used for summarizing demographic data. Quantitative variables were handled as is and were not categorized into groups. For continuous variables, a Student’s T-test and ANOVA test were used for comparison between groups. For categorical variables, a Chi-square test was used for comparison between groups. Only two-tailed tests were used and *p*-values less than 0.05 were considered significant. Missing values were not inferred.

## 3. Results

**Figure 1:**
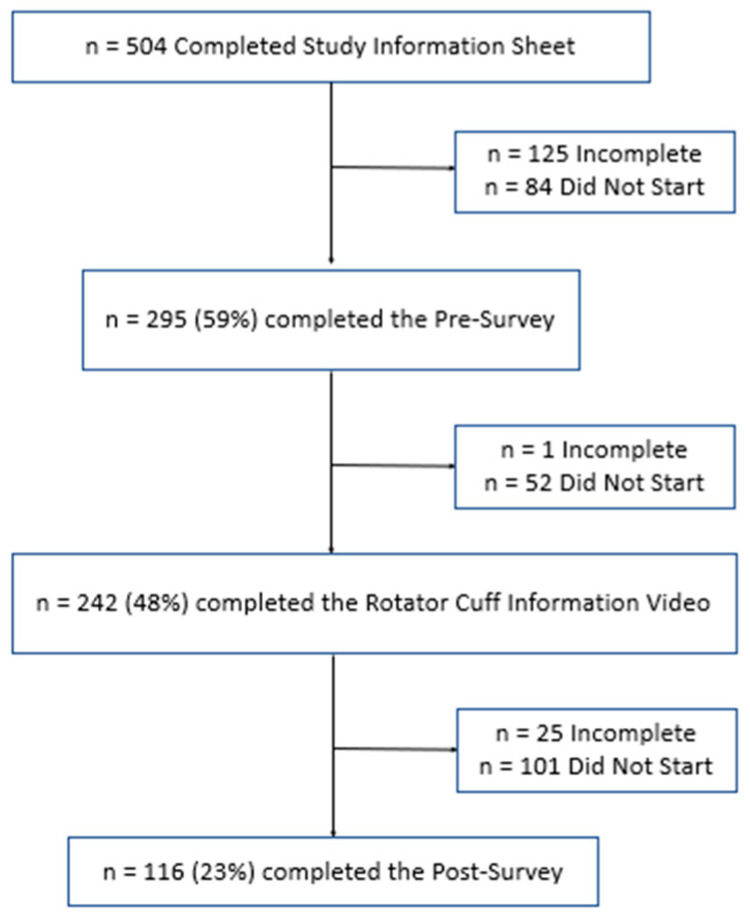
Low diagram for study participants.

A total of 504 participants completed the study information sheet, acknowledging that they were over the age of 18 and were participating voluntarily. Of these, 84 (16.7%) did not start the presurvey, 125 (24.8%) partially completed the presurvey, and 295 (58.5%) fully completed the presurvey. Next, participants were asked to watch an educational video about RCTs. Of the participants that completed the presurvey, 242 (48.0%) participants completed the educational intervention. Finally, participants were asked to complete the survey again to assess their new level of understanding. Of the participants who completed the rotator cuff video, 25 (5.0%) partially completed the postsurvey, and 116 (23.0%) fully completed both surveys. This is summarized below in Figure 1.

### 3.1. Demographic Characteristics of Respondents

Participant demographics are summarized in Table 2. A total of 382 participants (215 male and 167 female) completed the baseline survey, with males performing significantly higher than females (*p* < 0.001). The most common race/ethnicity to complete the survey was Caucasian (63.6%), followed by Asian (25.9%), Hispanic/Latino (3.9%), and Black/African American (3.4%). Participants were very well educated, with 8.9% having a doctoral degree, 27.0% having a master’s degree, 49.0% having an associate or bachelor’s degree, and 15.2% having a high school diploma or GED. Having a doctoral degree was correlated with a significantly higher score compared to individuals with other levels of education (*p* < 0.001). A total of 79.1% of participants had no work experience in the healthcare setting, and this was correlated with significantly lower scores when compared to individuals with healthcare experience (*p* < 0.001). Some 15.4% of participants had personal experience with RCTs and 23.6% of participants knew someone they knew who had an RCT. Participants with either personal experience or someone they knew who had an RCT performed significantly better than participants with no experience with RCTs (*p* < 0.001). Overall, participants did not rate their understanding of RCTs very highly: 33.0% rated themselves as having no knowledge, 23.0% rated themselves as having very little knowledge, 30.1% rated themselves as having some knowledge, 11.3% rated themselves as being very knowledgeable, and 2.6% rated themselves as experts. Participants’ self-rating of their knowledge of RCTs was significantly correlated with their performance (*p* < 0.001). The overall survey performance on each category for the pretest and posttest are summarized in Table 3, with posttest scores significantly improved in every category except the diagnosis and the risk of retears.

### 3.2. Anatomy, Function, and Epidemiology

During the baseline survey, a majority of participants (77.4%) understood that the primary function of the rotator cuff was the stability of the shoulder. However, only 47.3% answered that the rotator cuff was a tendon, while 40.5% believed that the rotator cuff was a ligament. This improved to 78.3% during the postsurvey (*p* < 0.001). Additionally, at baseline, only 20.5% of participants believed that the most common age group for rotator cuff tears (RCTs) involved populations older than 60. This increased to 64.5% during the postsurvey (*p* < 0.001).

### 3.3. Risk Factors for RCTs

At baseline, only 41.9% of participants believed that RCTs were usually the result of a significant injury. While this decreased to 37.3% during the postsurvey, this was not significant. However, at baseline, 85.5% believed that RCTs are painful and cause significant dysfunction, which improved to 41.6% during the postsurvey (*p* < 0.001). Moreover, only 22.2% believed that older adults with RCTs typically do not have any shoulder pain and dysfunction, which improved to 64.0% during the postsurvey (*p* < 0.001).

### 3.4. Diagnosis & Treatment Options

More than half of the participants (51.5%) correctly identified that the most definitive imaging test to diagnose RCTs is the MRI. While this improved to 56.9% at the postsurvey, this was not significant. When asked to rank the effectiveness of various treatment options for RCTs, participants ranked bone marrow and platelet-rich plasma (PRP) injections the lowest. They ranked anti-inflammatories (i.e., NSAIDs) and steroid (i.e., cortisone) injections the next highest. Finally, physical therapy and surgery were rated the highest. These ranking tiers were consistent even in the postsurvey.

At baseline, 65.1% of participants believed that RCTs would not heal on their own. Additionally, 62.4% of participants believed that almost all RCTs are able to be repaired with surgery, and 47.6% believed that most RCTs require surgery at some point. Finally, 65.7% believed that RCT surgery eliminates the risk of arthritis. During the postsurvey, 77.3% of participants recognized that most RCTs do not require surgery at some point (*p* < 0.001) and that RCT surgery does not eliminate the risk of arthritis (*p* < 0.05).

### 3.5. Surgical Management

At baseline, 68.0% of patients believed that, most commonly, RCT surgery involves multiple small poke-hole incisions and the use of a camera to repair torn muscle tendons. This improved to 88.1% during the postsurvey (*p* < 0.001). Moreover, 44.3% of participants expected to be discharged home the same day as surgery at baseline, which improved to 67.8% during the postsurvey (*p* < 0.001). The median pain level that was expected was six out of 10, and 39.0% of patients expected to receive at least one week of narcotic pain medication at baseline. This decreased to 20.3% of patients expecting to receive at least one week of narcotic pain medication during the postsurvey (*p* < 0.001).

### 3.6. Surgical Complications

The risk of infection was initially estimated to be 11.3 ± 6.0% on average but improved to 6.0 ± 6.8% (*p* < 0.001). The risk of nerve injury was initially estimated to be 12.9 ± 6.1% on average but improved to 6.3 ± 6.8% (*p* < 0.001). Similarly, the risk of stiffness decreased from an average of 17.1 ± 5.8% at baseline to 12.1 ± 5.7% after the procedure (*p* < 0.001). The risk of a retear was initially estimated to be 14.3 ± 6.2% on average and did not change with 13.6 ± 5.3% in the postsurvey.

### 3.7. Postsurgery Expectations

At baseline, 81.4% of participants expected one to recover at least 60% of normal function after RCT surgery, and 96.3% believed that physical therapy would be necessary. Participants anticipated one could return to activities of daily living (ADL) within 1–2 weeks (39.7%), light-duty work in 3–4 weeks (32.2%), and heavy-duty work in 5–7 months (23.7%). These expectations remained similar in the postsurvey.

## 4. Discussion

The general public demonstrated a prevalent lack of baseline knowledge regarding RCTs, with 56% reporting limited to no awareness. While certain aspects of understanding, such as the anatomy of the rotator cuff, may be considered less critical for immediate patient care, substantial knowledge deficits were identified in areas with direct clinical importance, notably risk factors (26.2%) and the postsurgery, return-to-activity timeline (35.9%).

Age-related susceptibility emerged as a critical point requiring emphasis, as enhancing public knowledge in this area is integral to promoting early detection and appropriate management, particularly within the aging population. This emphasis can empower patients to modify movement patterns, adopt proper techniques, and make positive lifestyle changes, ultimately reducing strain on the rotator cuff and minimizing the likelihood of developing tears [14,15]. Furthermore, our study highlighted the lack of understanding among participants regarding the crucial role of MRIs in diagnosing RCTs, even after the educational intervention, emphasizing the need to reinforce the importance of MRIs in the diagnostic process [16].

The study also explored participants’ perceptions of various treatment options for RCTs. Notably, bone marrow and PRP injections were ranked the lowest, while physical therapy and surgery were rated the highest. These findings resonate with current evidence-based treatment approaches that emphasize the role of physical therapy and surgical interventions in managing RCTs [17,18]. A noteworthy finding was that participants initially believed that RCTs do not resolve on their own, whereas in reality, many tears may become asymptomatic or compensated without requiring surgical intervention. This misconception was corrected significantly in the postsurvey, with a higher percentage of participants recognizing that not all RCTs require surgery and can potentially heal with nonsurgical interventions. This finding highlights the importance of patient education regarding the natural history of RCTs and the potential for conservative management in select cases [19].

Surgical management was also assessed in this study, including participants’ expectations regarding the duration of hospital stay and postoperative pain management. The significant increase in the postsurvey regarding the expectation of being discharged home the same day as surgery reflects an accurate understanding of the evolving surgical practices that promote early mobilization and outpatient care for most RCT cases [5]. Moreover, the decrease in the expectation of receiving at least one week of narcotic pain medication suggests an increased awareness of the importance of multimodal pain management strategies, which aim to minimize opioid use and promote faster recovery [20]. The study also assessed participants’ perceptions of the risks associated with RCT surgery. The significant improvements observed in the postsurvey regarding the estimated risks of infection, nerve injury, and stiffness indicate a more realistic understanding of the potential complications. Accurate knowledge of these risks enables patients to make informed decisions and actively participate in the management of their condition [21]. Managing patients’ expectations and providing accurate information about postsurgery outcomes is crucial for ensuring patient satisfaction and facilitating their recovery process. Participants in our study had specific expectations regarding the timeline for returning to various activities. For activities of daily living (ADL), the majority (39.7%) anticipated a return within 1–2 weeks, while for light-duty work, 32.2% expected a return within 3–4 weeks. Heavy-duty work was expected to resume in 5–7 months by 23.7% of participants. These expectations may be influenced by personal experiences, job demands, and the information available to patients. However, it is essential to emphasize that the timeline for returning to activities is highly individualized and depends on various factors, including the extent of the tear, surgical technique, and the individual’s progress during rehabilitation [22,23].

Finally, the implications of misconceptions in public understanding about RCTs are massive. For instance, this may lead to nonadherence to recommended treatments, as patients may believe that surgery is always necessary, neglecting conservative management options. Additionally, unrealistic expectations arising from these misconceptions could result in dissatisfaction with treatment outcomes and strain patient−provider relationships. Moreover, delays in seeking medical attention due to misconceptions about RCTs could lead to disease progression and poorer outcomes. Addressing these misconceptions through patient education is crucial, as it not only empowers patients to make informed decisions about their healthcare but also improves treatment adherence and overall quality of life. Thus, healthcare providers should prioritize patient education efforts to ensure that patients have accurate information about RCTs and their management options, ultimately optimizing patient outcomes.

In our exploration of public perceptions of rotator cuff tears, several limitations warrant consideration. First and foremost, the complexity of rotator cuff injuries manifests in diverse clinical scenarios, encompassing distinct etiologies, prognoses, and treatment modalities. While simplifying these concepts may be appropriate for a majority of patients, it runs the risk of confusion or inaccuracy for those with unique needs. Notably, variations in opinions on treatment approaches emerge not only among orthopedic physicians but also extend to sports physicians, primary care physicians, and physical therapists. The demographic profile of participants is also a limitation of our study. In comparison with the latest census data from 2020, there was a higher number of Asian (25.9%) participants and a lower number of Black/African American (3.4%) and Hispanic/Latino (3.9%) participants [24]. Furthermore, the study had a higher proportion of well-educated participants compared to the general population. The measures used in this study may not be as reliable for socioeconomically disadvantaged groups with lower levels of education. To mitigate this, all survey content was written at an 8th-grade level to ensure accessibility. We also did not collect data on the age of participants, which could be an important demographic variable to consider. Another limitation is that the survey questions were not formally validated, and there was no “I don’t know” response option provided, which may have affected the accuracy of participants’ answers and their level of confidence in them. However, we intentionally omitted the “I don’t know” option to understand better the prevalence of misconceptions about RCTs which was a weakness in the study methodology of other studies investigating the perceptions of orthopedic injuries [25,26,27,28]. We also did not differentiate between survey responses obtained from the senior author’s orthopaedic clinic and those obtained from online discussion platforms, which could have influenced the results. Furthermore, the length of the survey and the number of questions may have contributed to a lower response rate and an increased likelihood of incorrect answers as participants rushed to complete the survey. Finally, the study, as designed, may have limited immediate clinical implications, primarily affirming the initial hypothesis that general knowledge of rotator cuff issues is limited in the population. To address this limitation, future studies are recommended to adopt alternative methodologies, such as implementing two distinct patient groups—those undergoing conservative treatment and those undergoing surgery for rotator cuff injuries. In this proposed approach, one group would receive comprehensive and specific information about their condition beyond what healthcare providers typically offer, while the other would receive no additional information. Such a study design could shed light on the potential influence of enhanced information provision on treatment adherence, compliance levels, and patient satisfaction, offering valuable insights into the effectiveness of educational interventions in managing rotator cuff injuries. To broaden demographic inclusivity, future studies should employ sampling methods to reach a diverse population based on age, gender, geography, ethnicity, race, and culture. This could be accomplished by using zip code databases designed for such studies. To enhance subject learning, various educational media exist. A combination of written and video messages was used in this study, although an app-based tool may provide a more interactive experience.

## 5. Conclusions

To the best of our knowledge, this study represents the first examination of a specific population’s perception of rotator cuff tears (RCTs) and their treatment. Despite a skewed demographic profile, our findings are likely generalizable to the overall population. The study reveals a substantial knowledge gap among the public, particularly concerning risk factors and postsurgery and return-to-activity timelines, and demonstrates that a concise intervention significantly improved knowledge across all categories. Our research underscores variations in public understanding of RCTs, identifies prevalent misconceptions, and emphasizes the importance of targeted educational efforts. While the intervention positively impacted RCT knowledge, a crucial need persists for broader public education regarding evaluation and treatment. Future studies should delve deeper into patient perceptions to amplify public awareness and guide forthcoming research endeavors.

## Figures and Tables

**Table 1:** Categories of survey questions.

Question Category	Number of Questions
Demographics	6
Anatomy, Function, and Epidemiology	3
Risk Factors	3
Diagnosis & Treatment Options	11
Surgical Management	4
Surgical Risks	4
Post-Operative Expectations	5
Total	36

**Table 2:** Survey performance on the pretest and posttest stratified by participant.

Demographic Characteristic		n	Average Pretest Score	Significance	n	Average Posttest Score	Significance
**Gender**	Male	215 (56.3%)	50.3 ± 18.0%	***p* < 0.001**	63 (46.0%)	69.9 ± 24.4%	ns
	Female	167 (43.7%)	41.7 ± 16.8%		74 (54%)	65.6 ± 21.3%	
	Total	382			137		
**Race/Ethnicity**	American Indian or Alaska Native	8 (2.1%)	38.8 ± 11.9%	***p* < 0.001**	2 (1.5%)	52.9 ± 49.9%	***p* < 0.05**
	Asian	99 (25.9%)	36.3 ± 16.1%		31 (22.8%)	57.5 ± 23.4%	
	Black or African American	13 (3.4%)	38.2 ± 16.0%		7 (5.1%)	69.7 ± 30.1%	
	Hispanic or Latino	15 (3.9%)	54.7 ± 15.3%		4 (2.9%)	66.7 ± 24.6%	
	Native Hawaiian or Other Pacific Islander	4 (1.0%)	48.5 ± 13.0%		0	-	
	White	243 (63.6%)	50.8 ± 17.3%		92 (67.6%)	71.4 ± 20.7%	
	Total	382			136		
**Education Level**	High school or GED	58 (15.2%)	43.4 ± 16.0%	***p* < 0.001**	24 (17.5%)	63.7 ± 24.4%	ns
	Associate’s or Bachelor’s degree	187 (49.0%)	45.5 ± 16.6%		63 (46.0%)	68.4 ± 21.3%	
	Master’s degree	103 (27.0%)	45.1 ± 19.3%		35 (25.5%)	65.0 ± 21.3%	
	Doctoral degree	34 (8.9%)	61.7 ± 17.6%		15 (10.9%)	76.7 ± 28.5%	
	Total	382			137		
**Medical Profession**	Yes	80 (20.9%)	57.8 ± 17.1%	***p* < 0.001**	30 (21.9%)	71.0 ± 24.7%	ns
	No	302 (79.1%)	43.5 ± 17.0%		107 (78.1%)	66.6 ± 22.2%	
	Total	382			137		
**Personal Experience with RCTs**	Themselves	59 (15.4%)	56.0 ± 16.2%	***p* < 0.001**	14 (10.2%)	75.4 ± 22.9%	***p* < 0.05**
	Someone they know	90 (23.6%)	56.9 ± 18.8%		36 (26.3%)	74.4 ± 20.8%	
	None	233 (61.0%)	40.1 ± 14.9%		87 (63.5%)	63.5 ± 22.8%	
	Total	382			137		
**Self-Rated Understanding of RCTs**	No knowledge	126 (33.0%)	35.3 ± 12.0%	***p* < 0.001**	53 (38.7%)	58.5 ± 21.2%	***p* < 0.001**
	Very little knowledge	88 (23.0%)	44.2 ± 16.6%		28 (20.4%)	70.3 ± 22.0%	
	Some knowledge	115 (30.1%)	53.2 ± 15.8%		39 (28.5%)	74.2 ± 20.9%	
	Very knowledgeable	43 (11.3%)	60.6 ± 15.7%		13 (9.5%)	82.2 ± 17.3%	
	Expert	10 (2.6%)	71.4 ± 25.6%		4 (2.9%)	56.9 ± 38.0%	
	Total	382			137		

**Table 3:** Survey performance on the pretest and posttest stratified by question categories.

Question Category	n	Average Pretest Score	n	Average Posttest Score	Significance
Anatomy & Function	385	48.1 ± 27.7%	138	77.3 ± 30.7%	***p* < 0.001**
Injury Risk Factors	365	26.2 ± 27.7%	126	52.9 ± 35.3%	***p* < 0.001**
Diagnosis	361	51.5 ± 50.0%	123	56.9 ± 49.7%	ns
Treatment Options	332	54.0 ± 24.6%	119	67.4 ± 26.8%	***p* < 0.001**
Risk of Infection During Surgery	311	11.3 ± 6.0%	118	6.0 ± 6.8%	***p* < 0.001**
Risk of Nerve Injury During Surgery	311	12.9 ± 6.1%	118	6.3 ± 6.8%	***p* < 0.001**
Risk of Stiffness Following Surgery	311	17.1 ± 5.8%	118	12.1 ± 5.7%	***p* < 0.001**
Risk of Retear Following Surgery	311	14.3 ± 6.2%	118	13.6 ± 5.3%	ns
Surgical Management	300	43.8 ± 32.4%	118	70.9 ± 33.9%	***p* < 0.001**
Post-Operative Expectations	297	96.3 ± 18.9%	116	90.5 ± 29.4%	***p* < 0.05**
Return Timeline	295	35.9 ± 37.3%	116	67.7 ± 39.2%	***p* < 0.001**
Total	295	46.6 ± 18.0%	116	67.6 ± 22.8%	***p* < 0.001**

## Data Availability

Dataset available on request from the authors.

## Supplementary Materials

The following supporting information can be downloaded at: https://www.mdpi.com/article/10.3390/clinpract14030058/s1, File S1: Clinic Recruitment Letter, File S2: Online Recruitment Post, File S3: Survey Questionnaire, File S4: Rotator Cuff Tear Information Sheet.
