# Supplementary material for: Public Perceptions of Rotator Cuff Tears
Source: Clin Pract. 2024 Apr 25;14(3):729–38. doi: 10.3390/clinpract14030058 (PMC11130954; doi:10.3390/clinpract14030058)
Supplement: Supplementary file 1 [file clinpract-14-00058-s001.zip › File S1.pdf]

1. SURVEY Recruitment Letter (Posted Flyer in Clinics): Rotator Cuff Tear Study

Dear Patient,

We are studying rotator cuff tears and are particularly interested in how those in the public perceive these injuries. We are interested in your knowledge of these injuries as it may guide how we educate other patients, athletes, parents, and coaches.

A link to the survey is below and will take approximately 15-20 minutes to complete. I thank you in advance for your willingness to participate.

You may open the survey in your web browser by either scanning the QR code or clicking the link below:

[redacted]

https://redcap.link/[redacted]

For further information or questions regarding this study please feel free to contact us!

[redacted]
