# Supplementary material for: Public Perceptions of Rotator Cuff Tears
Source: Clin Pract. 2024 Apr 25;14(3):729–38. doi: 10.3390/clinpract14030058 (PMC11130954; doi:10.3390/clinpract14030058)
Supplement: Supplementary file 1 [file clinpract-14-00058-s001.zip › File S3.pdf]

Preintervention

Demographics

1) What is your sex?
- ◯ Male
- ◯ Female

2) What is your race/ethnicity?
- ◯ American Indian or Alaska Native
- ◯ Asian
- ◯ Black or African American
- ◯ Hispanic or Latino
- ◯ Native Hawaiian or Other Pacific Islander
- ◯ White

3) What is your highest education level?
- ◯ High school or GED
- ◯ Associate's or Bachelor's degree
- ◯ Master's degree
- ◯ Doctoral degree

4) Do you or did you work in a medical profession?
- ◯ Yes
- ◯ No

5) Have you or some you know ever been diagnosed with a rotator cuff tear or had a rotator cuff repair surgery?
- ◯ Myself
- ◯ Someone I know
- ◯ No

6) How would you rate your understanding of the rotator cuff?
- ◯ No knowledge
- ◯ Very little knowledge
- ◯ Some knowledge
- ◯ Very knowledgeable
- ◯ Expert

Anatomy & Function of The Rotator Cuff

7) The rotator cuff is a:

- ◯ Bone
- ◯ Ligament
- ◯ Tendon
- ◯ Nerve

8) What is the primary purpose of the rotator cuff?

- ◯ Bending your elbow
- ◯ Straightening out your knee
- ◯ Rotating your ankle
- ◯ Shoulder stability

9) Rotator cuff tears are most common in which age group?

- ◯ 10-20 years old
- ◯ 20-40 years old
- ◯ 40-60 years old
- ◯ >60 years old

Risk Factors for Rotator Cuff Injury

10) A rotator cuff tear is usually the result of a significant injury (ex: a fall, lifting a very heavy object, a car accident).

◯ True
◯ False

11) In general, rotator cuff tears are painful and cause significant dysfunction.

◯ True
◯ False

12) Older individuals who have rotator cuff tears typically do not have any shoulder pain or dysfunction.

◯ True
◯ False

Diagnosis

13) The most definitive imaging test to diagnose a rotator cuff tear is:

- ◯ X-ray
- ◯ CT scan
- ◯ Ultrasound
- ◯ MRI

How effective do you think the following therapies are at treating a rotator cuff tear (1 = not very effective & 10 = very effective)?

		1	2	3	4	5	6	7	8	9	10
14)	Physical Therapy	◯	◯	◯	◯	◯	◯	◯	◯	◯	◯
15)	Anti-inflammatories (i.e. NSAIDS)	◯	◯	◯	◯	◯	◯	◯	◯	◯	◯
16)	Steroid (cortisone) injection	◯	◯	◯	◯	◯	◯	◯	◯	◯	◯
17)	Bone marrow injection	◯	◯	◯	◯	◯	◯	◯	◯	◯	◯
18)	PRP injection	◯	◯	◯	◯	◯	◯	◯	◯	◯	◯
19)	Surgery	◯	◯	◯	◯	◯	◯	◯	◯	◯	◯

20) Rotator cuff tears can reliably heal on their own.
◯ True
◯ False

21) Almost all rotator cuff tears are able to be repaired with surgery.
◯ True
◯ False

22) Most rotator cuff injuries require surgery at some point in time.
◯ True
◯ False

23) Rotator cuff repair surgery eliminates the risk of arthritis following rotator cuff injury.
◯ True
◯ False

Risks of Surgery

24) What is the approximate risk for infection following rotator cuff repair surgery?

0% 25%

(Place a mark on the scale above)

25) What is the approximate risk for nerve injury following rotator cuff repair surgery?

0% 25%

(Place a mark on the scale above)

26) What is the approximate risk for stiffness at 6 months following rotator cuff repair surgery?

0% 25%

(Place a mark on the scale above)

27) What is the approximate risk for a retear at 6 months following rotator cuff repair surgery?

0% 25%

(Place a mark on the scale above)

Surgical Management

28) Most commonly, rotator cuff repair surgery involves:

- ◯ Multiple small "poke hole" incisions using a camera to repair the muscle tendons
- ◯ Multiple small "poke hole" incisions with cautery to cut the nerve
- ◯ A single large open incision with plates and screws to fix the fracture
- ◯ A single large open incision with a stent to repair the blood vessel

29) Following rotator cuff repair surgery, a patient can expect to:

- ◯ Be discharged from the hospital on the same day as surgery
- ◯ Spend 1 night in the hospital
- ◯ Spend 2-3 nights in the hospital
- ◯ Spend time in a rehabilitation facility

30) On a scale of 1-10, with 1 being minimal pain and 10 being extreme pain, what do you expect the pain from rotator cuff repair surgery to be?

1 ______________________________ 10

(Place a mark on the scale above)

31) After rotator cuff repair surgery, a patient can expect to need narcotic pain medications (ex: oxycodone, hydrocodone, etc) for _____ days.

- ◯ 1-2 days
- ◯ 3-7 days
- ◯ 7-14 days
- ◯ 14-21 days
- ◯ >21 days

Post-Surgical Expectations

32) After rotator cuff repair surgery, patients can expect their shoulder function to return to what percent (%) of normal:

- ◯ 0-10% normal
- ◯ 20-40% normal
- ◯ 40-60% normal
- ◯ 60-80% normal
- ◯ 80-99% normal
- ◯ 100% normal

33) Following rotator cuff repair surgery, physical therapy (working directly with a physical therapist) is usually necessary.

- ◯ True
- ◯ False

After rotator cuff repair surgery, how long can patients expect it will take before they can safely do the following tasks?

	1-2 weeks	3-4 weeks	1-3 months	3-5 months	5-7 months	7-9 months	9-12 months	> 1 year
34) Activities of daily living (i.e. feeding oneself, bathing, dressing) with assistance	○	○	○	○	○	○	○	○
35) Light duty work (i.e. desk job) that can be done with one hand	○	○	○	○	○	○	○	○
36) Heavy duty work (i.e. construction work), Weightlifting, Competitive sports	○	○	○	○	○	○	○	○
