# Supplementary material for: Public Perceptions of Rotator Cuff Tears
Source: Clin Pract. 2024 Apr 25;14(3):729–38. doi: 10.3390/clinpract14030058 (PMC11130954; doi:10.3390/clinpract14030058)
Supplement: Supplementary file 1 [file clinpract-14-00058-s001.zip › File S4.pdf]

Rotator Cuff Information

What is the Rotator Cuff?

The Rotator Cuff refers to a group of muscles and their tendons that surround your shoulder joint. A tendon attaches a muscle to bone whereas a ligament attaches a bone to another bone. The primary purpose of the rotator cuff is to provide stability to the shoulder joint while allowing you to lift your shoulder and rotate your arm.

What is a Rotator Cuff Injury?

Rotator cuff injuries can include:
Full thickness tear where the tendon is completely torn
Partial thickness tear where the tendon is incompletely torn
Impingement where the tendon is being pinched as the muscles are moving

What causes a Rotator Cuff Injury?

Sudden Injury
Traumatic fall, Car accident, Lifting heavy weights incorrectly

Chronic Injury
Sports with repetitive overhead arm motions (i.e. baseball, tennis, volleyball, swimming)
Jobs with routine chores (i.e. painters, construction workers, movers)
General wear and tear with aging

Although rotator cuff injuries can happen due to an acute event, they most commonly occur in older adults (> 60 years) with minimal symptoms

Symptoms of a Rotator Cuff Injury

Those with a Rotator Cuff Injury may or may not feel: pain, limited ability to move their arm, muscle weakness in their arm, grating or crackling sensation with movement.

In fact, the majority of rotator cuff injuries present without symptoms (66%)

Diagnosing a Rotator Cuff Injury

Requires a combination of the patient's history, physical examination, imaging (X-rays or MRI). These tests are not 100% accurate, making diagnosis sometimes difficult.

Do you need Rotator Cuff Surgery?

While Rotator Cuff Injuries cannot reliably heal on their own, not all injuries require surgery. In fact, In most patients, a Rotator Cuff Injury is usually first treated without surgery and is effective in 80% of patients.

It involves rest and ice, anti-inflammatories (i.e. ibuprofen), and steroid (i.e. cortisone) injections. Afterwards, physical therapy is used to strengthen surrounding muscles to compensate for the injured rotator cuff.

What happens during Surgery?

Surgery is most commonly done arthroscopically with small pokeholes in the skin rather than a big incision
Step 1: multiple small pokeholes are made to insert a camera and other tools for the surgeon
Step 2: tendons are re-attached and secured to the bone using small rivets (suture anchors)
Step 3: stitches are attached to the anchors, which tie the tendon back to the bone

Rotator Cuff Surgery Misconceptions and Complications

Surgery CANNOT fix every Rotator Cuff Injury
Most Rotator Cuff Injuries DO NOT require surgery
Surgery DOES NOT eliminate the risk of arthritis (stiffness) following repair

Surgery IS NOT without risks
Infection (1-2%)
Nerve Injury (1-2%)

Post-operative stiffness 6 months out from surgery (8-10%)
Retearing of the Rotator Cuff 6 months out from surgery (16%)

Expectations after Surgery

After surgery, patients can expect to be discharged from the hospital the same day with 1-2 days of narcotic pain medication (i.e. oxycodone, etc.). Patients are usually advised to rest for a few weeks, before starting physical therapy to recover strength and mobility of their arm.

1-2 Weeks: Return to activities of daily living (bathing or showering, dressing, getting in and out of bed, walking, eating)
3-4 Weeks: Return to light duty work
Several Months: Return to heavy duty work, weightlifting, competitive sports
