# Supplementary figures and images for: Public Perceptions of Rotator Cuff Tears
Source: Clin Pract. 2024 Apr 25;14(3):729–38. doi: 10.3390/clinpract14030058 (PMC11130954; doi:10.3390/clinpract14030058)

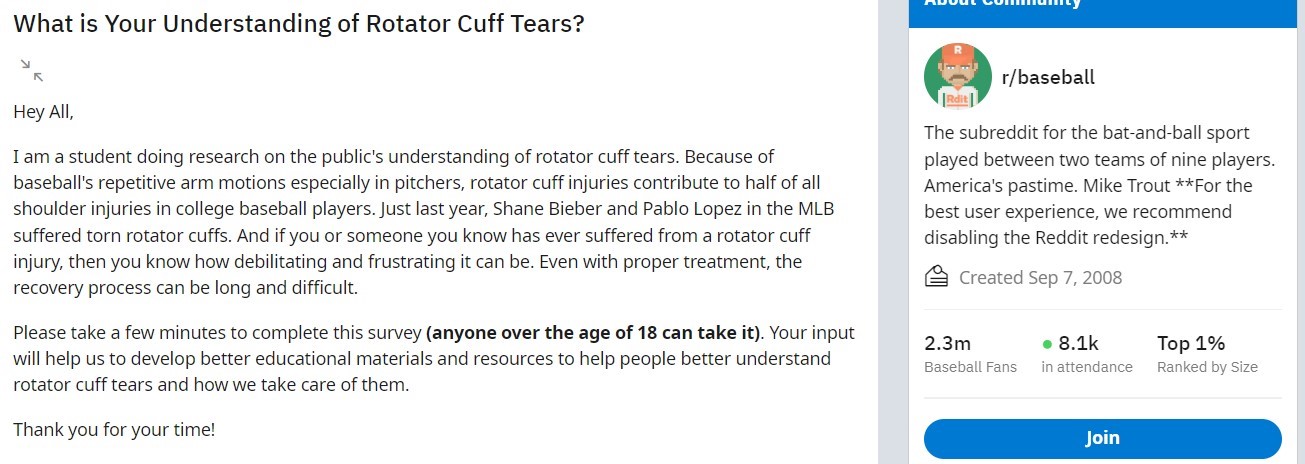

Supplement: Supplementary file 1 [file clinpract-14-00058-s001.zip › File S2.jpg]
